# Ultra strong pyroprotein fibres with long-range ordering

**DOI:** 10.1038/s41467-017-00132-3

**Published:** 2017-07-13

**Authors:** Se Youn Cho, Young Soo Yun, Dawon Jang, Jun Woo Jeon, Byung Hoon Kim, Sungho Lee, Hyoung-Joon Jin

**Affiliations:** 1 Department of Polymer Science and Engineering, Inha University, Incheon, 402-751 Korea; 2 Department of Chemical Engineering, Kangwon National University, Samcheok, 245-711 Korea; 3 Carbon Composite Materials Research Center, Institute of Advanced Composite Materials, Korea Institute of Science and Technology, 92 Chudong-ro, Bongdong-eup, Wanju-gun, Jeollabuk-do, 55324 Republic of Korea; 4 Department of Nano Material Engineering, Korea University of Science and Technology, 217 Gajeong-ro, Yuseong-gu, Daejeon, 34113 Republic of Korea; 5 Department of Physics, Incheon National University, Incheon, 406-772 South Korea

## Abstract

Silks are protein-based natural structured materials with an unusual combination of high strength and elongation. Their unique microstructural features composed of hard β-sheet crystals aligned within a soft amorphous region lead to the robust properties of silks. Herein we report a large enhancement in the intrinsic properties of silk through the transformation of the basic building blocks into a poly-hexagonal carbon structure by a simple heat treatment with axial stretching. The carbon clusters originating from the β-sheet retain the preferred orientation along the fibre axis, resulting in a long-range-ordered graphitic structure by increasing heat-treatment temperatures and leading improvements in mechanical properties with a maximum strength and modulus up to ∼2.6 and ∼470 GPa, respectively, almost four and thirty times surpassing those of raw silk. Moreover, the formation of *sp* ^2^ carbon configurations induce a significant change in the electrical properties (e.g. an electrical conductivity up to 4.37 × 10^3^ S cm^−1^).

## Abstract (web summary)

The mechanical properties of silk are determined by tight stacks of sheet-like peptide crystals distributed in amorphous regions. Here, the authors heat and stretch silk fibres to align these crystal into a long range ordered carbon structure and dramatically enhance the silk strength.

## Introduction

Natural materials consisting of simple components with poor intrinsic mechanical properties sometimes possess superb properties and perform their own function through an exquisite microstructure, as illustrated by the shock-absorbing systems of woodpeckers, the adhesion properties of gecko foot-hairs and the toughness of nacre^1–5^. Proteins, one of the most abundant and versatile macromolecules in living systems, are biopolymers built of amino acids by peptide linkages. Their own mechanical properties and biological functions highly influenced by a microstructure determined by a sequence of amino acids^6^. Silks produced by arthropods such as silkworms and spiders are the representative fibrous proteins mainly composed of glycine and alanine. The high contents of these two amino acids result in highly conserved amino acid repeat units such as poly-(Gly-Ala) and poly-Ala domains in the primary sequence^7^. These repeated peptide domains promote the polypeptides to construct a unique secondary structure, β-sheet conformations, through assemblies of numerous inter-/intra-chain hydrogen bonds. Moreover, the short side chains of the repeat units (a hydrogen atom for glycine and a methyl group for alanine) lead to tight stacks of the sheet structures, termed β-sheet crystallites with a rectangular coordinate system^8, 9^. While typical protein chains linked by peptide bonding are so flexible that diverse molecular conformations can be formed, the β-sheet crystals formed by the strong supramolecular interactions reveal rigid and robust material characteristics with a high modulus and strength of up to 10–28 GPa and 0.6–1 GPa, respectively^10–14^. Therefore, the contents and distributions of the stiff β-sheet crystals in the amorphous region play a decisive role in the mechanical properties of silk materials. In particular, *Bombyx mori* (*B. mori*, domesticated) silkworm silk possesses a high content of β-sheet crystals (~0.3–0.6) arranged along the silk fibre axis *via* a liquid-to-solid transition of a highly concentrated silk protein solution stored in a state of liquid-crystal intermediates in the silkworm gland^15–20^. Consequently, silkworm silk achieves superb mechanical properties for its function of protection from environmental enemies before moulting. This unique-engineered structure has guided new directions for a state-of-the-art material design in modern scientific and technological fields^21–26^. As another interesting phenomenon, the β-sheet structures of *B. mori* silk can be chemically transformed into poly-hexagonal carbon structures, pyroproteins, through simple heating^27^.Although full comprehension of the graphitisation mechanism of carbon materials prepared from organic precursors still remains challenging, it is known that the nanometric unsaturated or aromatic building blocks formed after thermal degradation of the main backbone develop into ordered carbon structures through several steps, such as elimination of heteroatoms, condensation and dehydration following heating up to 3000 °C. Therefore, the parallel alignment and stacking of the poly-hexagonal carbon building blocks serve as criteria for constructing a long-range-ordered graphitic structure^28–30^. Typical graphitisable carbon precursors such as petroleum and coal pitches pass through a viscoelastic stage termed the mesophase, wherein a local orientation of polyaromatic molecules can be formed by a spinning process. By further heating above 2000 °C, the arranged molecules develop into large graphene layer stacks^29, 30^. However, in spite of the highly arranged structure of the β-sheet crystals in silk, the resulting carbon clusters of pyroproteins reveal no oriented structure. By further heat treatment up to 2800 °C, they develop into a pseudo-graphitic structure similar to a typical non-graphitising carbon material^27, 31^.

Herein, we applied a loading stress during the pyrolysis process of silk at 350 °C, at which their disordering occurs owing to the core chemical and structural changes. This simple process resulted in obviously arranged microstructure of the resulting samples. And by further heating process up to 2800 °C, the long-range-ordered carbon structures were developed, leading improvements in mechanical properties with a maximum strength and modulus up to ~2.6 and ~470 GPa, respectively, and the electrical properties (e.g. an electrical conductivity up to 4.37 × 10^3^ S cm^−1^).

## Results

### Pyrolysis of silk fibres

**Fig. 1:**
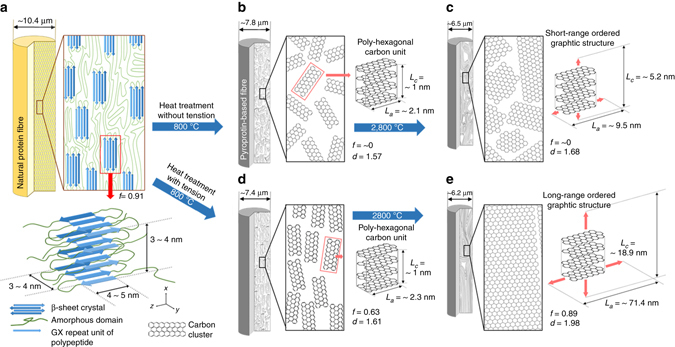
Schematic of long-range-ordered pyroprotein-based fibre. **a** The structure of silk composed of highly aligned β-sheet crystals organised by the self-assembly of GX repeat units through a number of inter-/intra-chain hydrogen bonds and surrounding amorphous domains consisting of non-repetitive peptide chains. **b** At temperatures in excess of 800 °C, disordered poly-hexagonal carbon units are formed by the pyrolysis of poly-peptide molecules. **c** Following heating to 2800 °C, the disordered poly-hexagonal carbon units developed into pseudo-graphitic domains. **d** By axial stretching, pyroprotein-based fibres with well-arranged poly-hexagonal carbon units along the fibre axis are formed by heating to 800 °C. **e** Long-range-ordered graphitic structures evolve following heating to 2800 °C

*B. mori* silk is composed of amorphous chains and β-sheet crystallites aligned parallel to the fibre axis with high degree of preferred orientation over 0.9 (see Fig. 1a, Supplementary Fig. 1 and Supplementary Note 1). However, during the pyrolysis process, the microstructure of silk fibres is restructured into a carbonaceous form through chain scission and the emission of flue gases confirmed by the shrinkage of silk fibres (see Supplementary Figs. 2, 3 and Supplementary Note 2). In particular, in the region of 250 ~ 350 °C, major shrinkage (~17 and 26% reductions in longitudinal and transverse directions, respectively) and mass loss (~50 wt%, which is ~0% of the overall mass loss) occur, leading to distortion of their own arranged structure (Supplementary Fig. 4a and b). A further increase in the heat-treatment temperatures (HTT) brings about the formation and growth of a randomly oriented graphitic structure (Fig. 1b and c). However, after applying a loading stress of 7 MPa during the pyrolysis process of silk at 350 °C with adequate dwell time of 3 h, the microstructure of pyroproteins somewhat preserves the inherent alignment even after transformation into the carbon-based structure (Supplementary Fig. 4c and d). Namely, the poly-hexagonal carbon structures have been formed from the β-sheet crystallites with maintaining the parallel alignment. In order to confirm whether the aligned carbon structure can develop into a large graphitic structure according to the general graphitisation mechanism (Fig. 1d and e), the pyroprotein fibres were submitted to further heating process up to 2800 °C. The corresponding samples with the loading stress are denoted according to the final HTTs, e.g. SSF800, SSF1200, etc., and the reference samples prepared by simple heating without the axial stretching are also denoted as SF800, SF1200, etc.

**Fig. 2:**
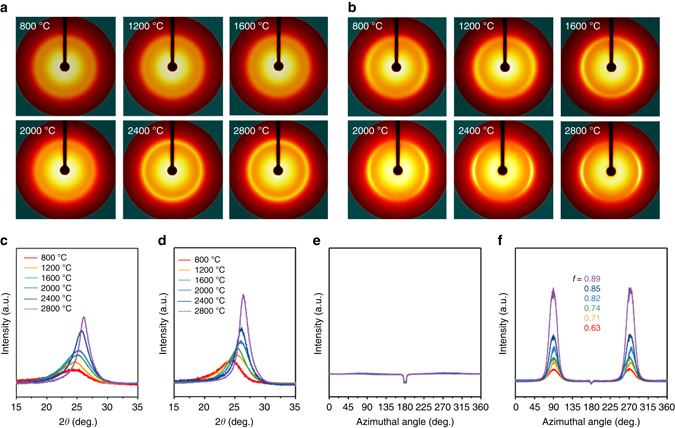
X-ray diffraction profiles of pyroprotein-based fibres by heating to 2800 °C. **a**,**b** WAXD patterns, **c**,**d**, 1D radial integration profiles of entire 2D patterns and **e**,**f**, 1D azimuthal intensity profiles of the radially integrated (002) peak with Gaussian fits for silk-fibre samples treated at different temperatures with and without axial stretching, respectively

As a result of the transformation, graphitic (002) diffraction peaks are observed at around 2*θ* = 24 – 26° for both SSF and SF samples in the wide angle X-ray diffraction (WAXD) patterns^32^ (Fig. 2). According to the increasing HTT, the crystalline reflections become sharper and shift to the high-angle region, indicating growth of the crystalline carbon domain(along the *c* axis) and the formation of denser structures for both SSFs and SFs (Fig. 2a–d, Supplementary Fig. 5 and Supplementary Note 3). However, compared to the SF samples exhibiting no recognisable peaks in the azimuthal intensity profiles (Fig. 2e), the SSF samples show significantly strong peaks in the azimuthal angle, representing a well-oriented crystalline structure along the fibre axis (Fig. 2f). Furthermore, the relative crystalline orientation becomes more pronounced with increasing HTT, identified by the increase in the degree of preferred orientation from 0.63 for SSF800 to 0.89 for SSF2800. Namely, through the axial stretching of silk fibres at 350 °C, a well-oriented carbon structure is induced for SSFs compared with the disordered carbon structures for SF samples. Moreover, the arranged carbon structure becomes more prevalent with increasing HTT.

**Fig. 3:**
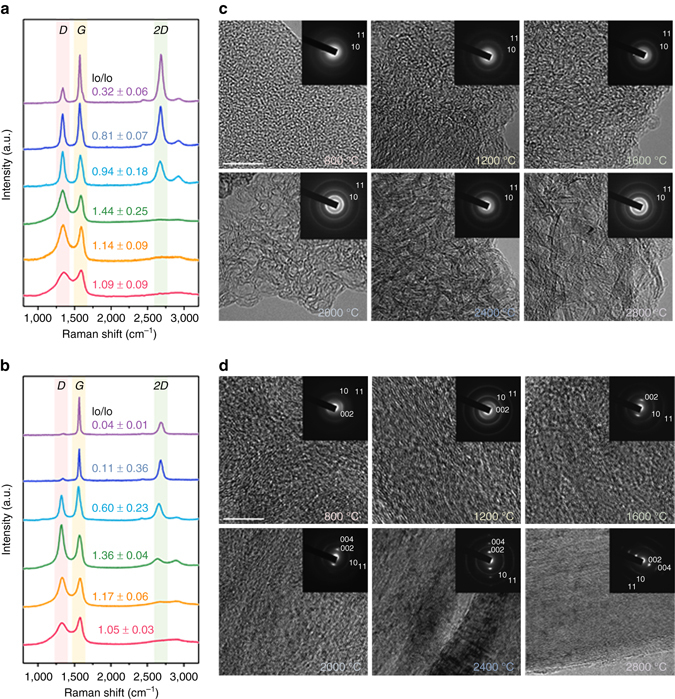
Heat-treatment temperature dependent microstructural characteristics of pyroprotein-based fibres by heating to 2800 °C. **a**,**b** Raman spectra, and **c**,**d** TEM and selected area diffraction patterns of the pyroprotein-derived fibres with and without axial stretching, respectively, as a function of the HTT. The scale bars in the *top-left* image in **c** and **d** represent 10 nm

More specific examinations of the microstructural development of both SF and SSF samples were carried out using Raman spectroscopy and field emission-transmission electron microscopy (FE-TEM). From the Raman spectra (Fig. 3a and b), it is observed that the carbon structure of the pyroprotein fibres develop through the two stage transition similar to the that of other polymeric carbon precursors from 800 to 2800 °C^29, 34^. Below 1600 °C, the two broad carbon crystallite peaks, *D* and *G* bands (manifesting around 1350 and 1580 cm^−1^, respectively) are presented overlapping with other bands associated with disordered carbon structures. In this region, both SF and SSF samples showed the slight increase of the intensity ratios of the *D* to *G* bands (*I*_D_/*I*_G_), indicating the slight decrease of the crystal size due to depolymerisation of pyroproteins^33,34^. However, even though both SF and SSF samples showed the development of a three-dimensional-ordered carbon structure identified by the formation of a 2*D* band (at around 2690 cm^−1^)^35, 36^, the microstructures evolve in a completely different manner above 1600 °C. While the average lateral carbon crystal size (*L*_a_) of the SSF samples, which were calculated from the intensity ratios of the *D* to *G* bands (*I*_D_/*I*_G_), rapidly increases from 1600 °C (*L*_a_ = 1.79 nm) to 2800 °C (*L*_a_ = 102.67 nm), the carbon crystallites in the SF samples slightly increase for the same HTTs (*L*_a_ = 1.75 for SF1600 and *L*_a_ = 15.14 for SF2800, see Supplementary Figs. 6–8 and Supplementary Note 4).The structural evolutions of the SF and SSF samples characterised by FE-TEM are in agreement with the XRD and Raman results (see Fig. 3c and d, and Supplementary Note 5).While the SF samples show a disordered crystalline structure without any long-range carbon ordering throughout all temperature ranges, the SSF samples exhibit a well-aligned carbon microstructure parallel to the fibre axis, even at a low temperature of 800 °C. As the HTT increases to 2800 °C, a long-range-ordered graphitic structure with an interlayer spacing of *d*_002_ = 0.34 nm is observed for SSF2800 (Supplementary Fig. 9), whereas disordered configurations are revealed for SF2800, as shown in the FE-TEM images. However, it is noteworthy that in spite of the pyrolysis behaviour of the silk exhibiting no plastic stage, SSF2800 shows a long-range-ordered graphitic structure comparable to that of a graphitisable carbon precursor. This result suggests that the β-sheet crystals are transformed into a poly-hexagonal carbon structure while maintaining their intrinsic arrangement along the fibre axis under the axial stress.

### Mechanical and electrical properties after pyrolysis

**Fig. 4:**
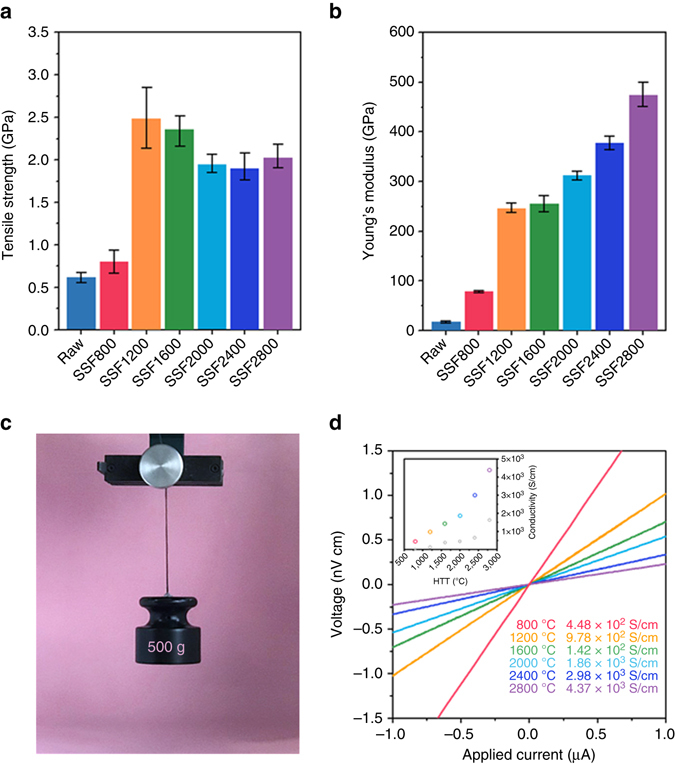
Mechanical and electrical properties of the pyroprotein-based fibres as a function of the heat-treatment temperature. **a** Tensile strength and **b** Young’s modulus of the pyroprotein-derived fibre samples treated at different temperatures with axial stretching, estimated by a mechanical tester for a single fibre (*n* = 10 for each group). The error bars denote standard deviations of the mean. **c** Photograph of the SSF1200 bundle enduring 0.5 kg of loading weight. **d** *V–I* curves of the pyroprotein-derived fibres for various HTTs and (inset) the conductivity obtained from the inverse slope of *V–I* curves as a function of the HTT

The progression of the mechanical and electrical properties according to the chemical transition into a rigid carbon structure for SSF samples was investigated as a function of the HTT (Fig. 4, Supplementary Table 2). The SSF samples show a significant enhancement in the tensile strength from 0.6 ± 0.1 GPa for raw silk to 2.6 ± 0.3 GPa for SSF1200 with linear elastic behaviour starting at zero strain until the failure strain. However, further increase in the HTT up to 2800 °C does not contribute to improvement in the tensile strength (Fig. 4a). The initial increase in the strength of the SSF samples by increasing the HTT up to 1200 °C can be explained by synergistic effects: partial restoration of the intrinsic mechanical properties of the basic components, poly-hexagonal carbon units, by thermo-healing of defective carbon domains through the elimination of non-carbon elements and the three-dimensional inter-layer forces resulting from the covalent bonding between the remaining *sp* ^3^ carbon atoms (see Supplementary Figs. 10–12, Supplementary Tables 3 and 4 and Supplementary Note 6). In contrast, further heat treatment at temperature over 1600 °C results in the growth of the *sp* ^2^ carbon domains indicating the diminution of the *sp* ^3^ carbon content, inevitably leading to a decrease in the tensile strength^37^. In contrast, the Young’s modulus of the SSF samples tends to increase gradually as the HTT increases to 2800 °C, which is closely linked to the graphitic microstructure. The increasing HTT of the pyroprotein-based fibres leads to the continuous development in both size and preferred alignment of graphitic structures and the thermo-healing of *sp* ^2^ lateral carbon layers. Consequently, SSF2800 with a long-range-ordered graphitic structure exhibits the maximum Young’s modulus of 470 ± 30 GPa which is almost thirty-times higher than the average modulus for raw silk (Fig. 4b). These strong mechanical properties of SSF can be recognised at a glance from the photograph as shown in Fig. 4c.

In addition, the electrical conductivities of the SSF samples are also highly affected by the evolution of the graphitic structures. Figure 3d shows the voltage–current (*V*–*I*) characteristics of the SSF samples at different HTTs measured by a typical four-probe method. As the HTT increases, the slope of the linear and symmetric curves tends to decrease, indicating a decrease in the resistivity through the development of carbon clusters generating a well-developed conducting pathway. Compared to the electrical conductivities of the SF samples (inset of Fig. 4d, *grey circles*), the SSF samples show much higher electric conductivities by almost an order of magnitude, which also supports the increase in the ordering of the carbon microstructure for SSF samples. SSF2800 finally exhibits a high electrical conductivity up to 4.37 × 10^3^ S cm^−1^.

In this study, we demonstrated that the intrinsic properties of a natural fibre, silk, limited by its basic components, protein molecules, can be overcome to become one of the high-performance fibre in the materials field owing to a unique microstructure with a reconstructed chemical structure. The β-sheet structures of silk are transformed into more rigid and stronger aromatic poly-hexagonal carbon units while retaining their parallel arrangement along the fibre axis through a simple heating with axial stretching. Moreover, the fabricated pyroproteins become more ordered by further heat treatment to 2800 °C, resulting in enhanced mechanical and electrical properties. SSF1200 with *sp* ^2^- and *sp* ^3^-hybridised carbon structures exhibits an ultimate tensile strength up to ~2.6 GPa. In addition,SSF2800 with a long-range-ordered graphitic structure displays more rigid mechanical and highly conductive properties, resulting in a Young’s modulus up to ~470 GPa and high-electrical conductivity up to 4.37 × 10^3^ S cm^−1^.

## Methods

### Materials

Cocoons from *B. mori* silkworms were purchased from the Uljin Farm, South Korea. In order to extract the glue-like sericin proteins and impurities, silkworm cocoons were boiled for 25 min in an aqueous solution of 0.02-M Na_2_CO_3_ (99%; OCI Co.) and washed with deionised water several times. Followed by drying at room temperature for 3 days, the resulting fibrous material (silk fibres) was used in further experiments.

### Heat treatments with axial stretching

With a constant loading stress of ~7 MPa by suspending a graphite ball of 20 g, ~350 strands of silk fibres were heated to 150 °C and dwelled at that temperature for 1 h in order to remove absorbed water molecules. After isothermal pyrolysis for 3 h at 350 °C, the samples were heated to the desired temperature (800, 1200, 1600, 2000, 2400 and 2800 °C) in an argon atmosphere (minimum purity, 99.999%; gas flow, 100 cm^3^ min^−1^) at a rate of 5 °C min^−1^. The samples with HTTs up to 1600 °C were carbonised in an alumina furnace and a graphitisation furnace (ThermVac, Korea) was used to achieve temperatures higher than 2000 °C. Once the desired temperature was reached, the samples were maintained at this temperature for 1 h and cooled to room temperature in the same argon flow.

### Field emission-transmission electron microscopy

FE-TEM measurements were carried out using a JEM2100F (JEOL, Japan) microscope equipped with an energy-dispersive X-ray spectrometer. The finely grounded samples were dispersed in ethanol, and a drop of the resulting dispersion was deposited onto a copper TEM grid.

### Raman spectroscopy

Raman spectra were recorded with a Raman spectrometer (LabRAM HR, Horiba, France) using a laser excitation wavelength of 514.5 nm (giving a photon energy of 2.41 eV). After fixing the sample on a slide glass using adhesive tape, Raman spectra were obtained over the range 800–3500 cm^−1^ under ambient conditions with a power of 16 mW. The laser beam was focused using a ×100 objective lens, resulting in a spot size of ~1 mm in diameter. The acquisition time was 10 s, and the number of circulations was three.

### Wide angle X-ray diffraction

WAXD patterns were acquired using a D8 Discover X-ray diffractometer equipped with a CuKa radiation (*λ* = 0.154018 nm) and a Vantec 500 detector (Bruker, Germany). Specimens of fibre bundles were placed perpendicular to the beam with a sample-to-detector distance of 8.3 cm and exposed to the X-ray beam for 100 s.

### Degree of preferred orientation

The degree of preferred orientation of the graphene layers, *f*, along the fibre axis was obtained from an azimuthal integration at the fixed Bragg position of the (002) reflection, calculated by the formula:

1 $$f = \left( {180 - {Z^ \circ }} \right){\rm{/}}180$$

where *Z* is the full width at half maximum of the azimuthal intensity.

### X-ray photoelectron spectroscopy

X-ray photoelectron spectroscopy data were collected by aPHI 5700 ESCA (Physical Electronics Inc., USA) with monochromatic Al-Kα radiation (*hv* = 1486.6 eV) in order to examine the type of chemical bond of nitrogen and the chemical composition on the surface of samples.

### Elemental analysis

An elemental analysis (Flash 2000, Thermo Scientific, USA) was performed to measure the amounts of C, H, N and O in the bulk samples. The elements of C, H and N were analysed using oxygen and helium gas at 900 °C for 720 s. Oxygen was analysed using helium gas at 1060 °C for 500 s.

### Mechanical properties

The mechanical properties of the raw silk fibres and SSF samples were examined by a mechanical tester for a single fibre (FAVIMAT+, Textechno, Germany) with a test speed of 5 mm min^−1^. The Gauge length was 25 mm, and 10 specimens were tested for each of the samples.

### Electrical conductivity

The electrical conductivity of SSF samples was investigated using a conventional four-probe method. A single fibre of SSF samples was attached to gold wires (0.01 inches in diameter) using silver paint (DuPont 4929N). The *I–V* characteristics were measured using a current source (6220 DC current source; Keithley) and a nanovoltmeter (2182 nanovoltmeter; Keithley). The current was applied to SSF samples from −1 to 1 μA through dual sweep. The step was 0.01 μA and each delay time is 1 s. The electrical conductivity was obtained from the inverse slope of *V–I* curves.

### Data availability

The authors declare that the data supporting the findings of this study are available within the article and its Supplementary information files. All other relevant data supporting the findings of this study are available from the corresponding author on request.

## Electronic supplementary material

Supplementary Information
