# Supplementary material for: Ultra strong pyroprotein fibres with long-range ordering
Source: Nat Commun. 2017 Jul 13;8:74. doi: 10.1038/s41467-017-00132-3 (PMC5509745; doi:10.1038/s41467-017-00132-3)
Supplement: Supplementary file 1 — Supplementary Information [file 41467_2017_132_MOESM1_ESM.pdf]

File name: Supplementary Information
Description: Supplementary Figures, Supplementary Tables, Supplementary Notes and Supplementary References

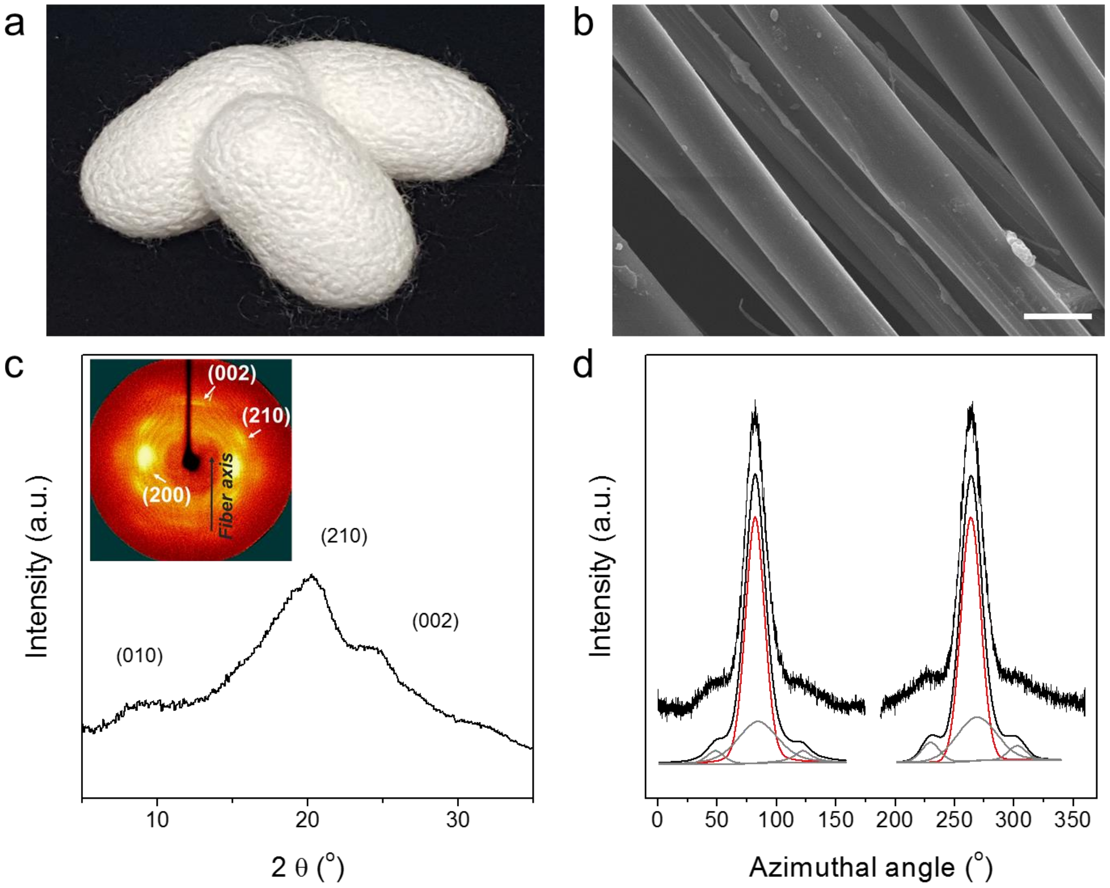

Supplementary Fig. 1. Microstructure of silk. (**a**) Optical image of cocoons from the *B. mori* silkworm. (**b**) FE-SEM images of silk fibroins extracted from the *B. mori* silkworm cocoons by degumming process. Scale bar, 10 μm. (**c**) Wide-angle XRD patterns of silk fibroin with the reflections corresponding to the (200), (210) and (002) planes of the protein β-sheet structure. (**d**) 1D azimuthal intensity profiles of the (200) peak for silk fibroin with a degree of preferred orientation of 0.91. The peaks are fitted with the sums of Gaussian curves corresponding to crystalline (red) and amorphous (grey) distributions.

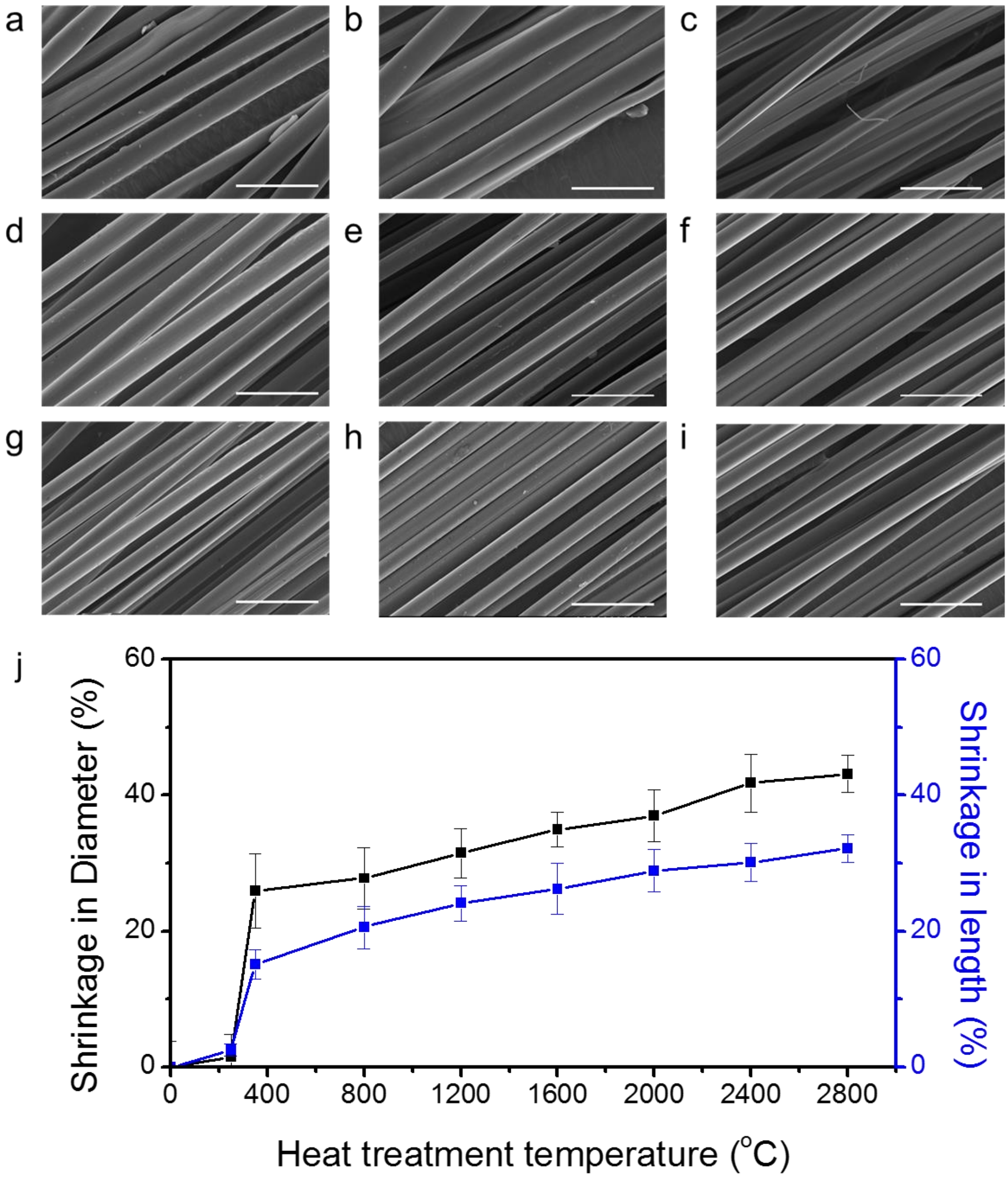

Supplementary Fig. 2. Thermal shrinkage of the silk fibres as a function of the temperature. FE-SEM images of (**a**) raw silk fibroin fibres and silk fibres heated to (**b**) 300 ºC, (**c**) 400 ºC, (**d**) 800 ºC, (**e**) 1,200 ºC, (**f**) 1,600 ºC, (**g**) 2,000 ºC, (**h**) 2,400 ºC, and (**i**) 2,800 ºC. Scale bars, 30 μm for panels a-c and 20 μm for panels d-i. (**j**) Thermal shrinkage in the longitudinal and transverse direction of the silk fibroin fibres as a function of the HTT, obtained from the SEM images (n=20 for each sample). The error bars denote standard deviations of the mean.

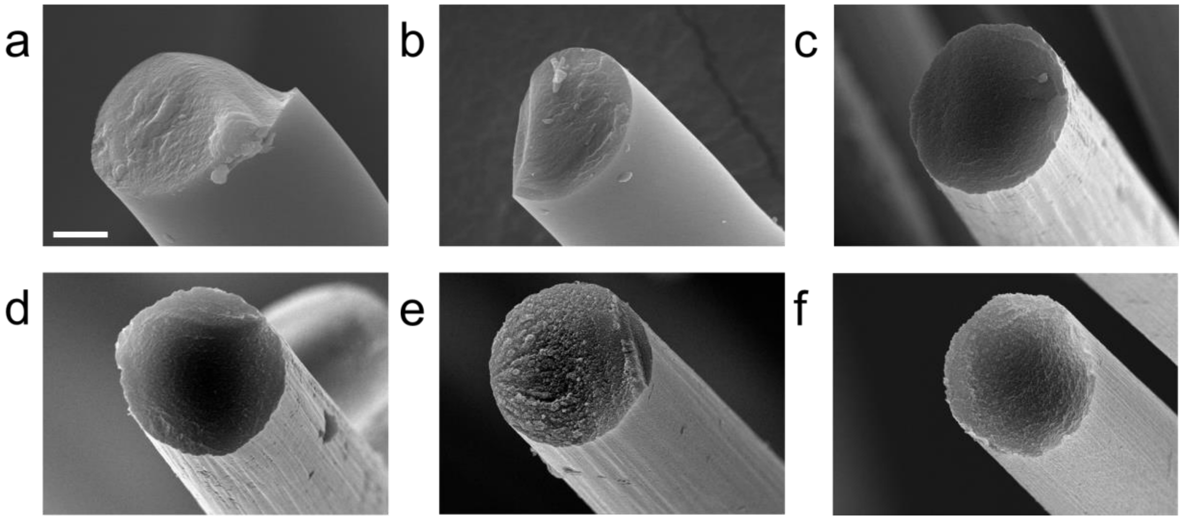

Supplementary Fig. 3. FESEM images of cross section of silk fibres heated to (a) 800 ºC, (b) 1,200 ºC, (c) 1,600 ºC, (d) 2,000 ºC, (e) 2,400 ºC, and (f) 2,800 ºC. The scale bar in panel a represents 2 μm and is applicable to panels b-f.

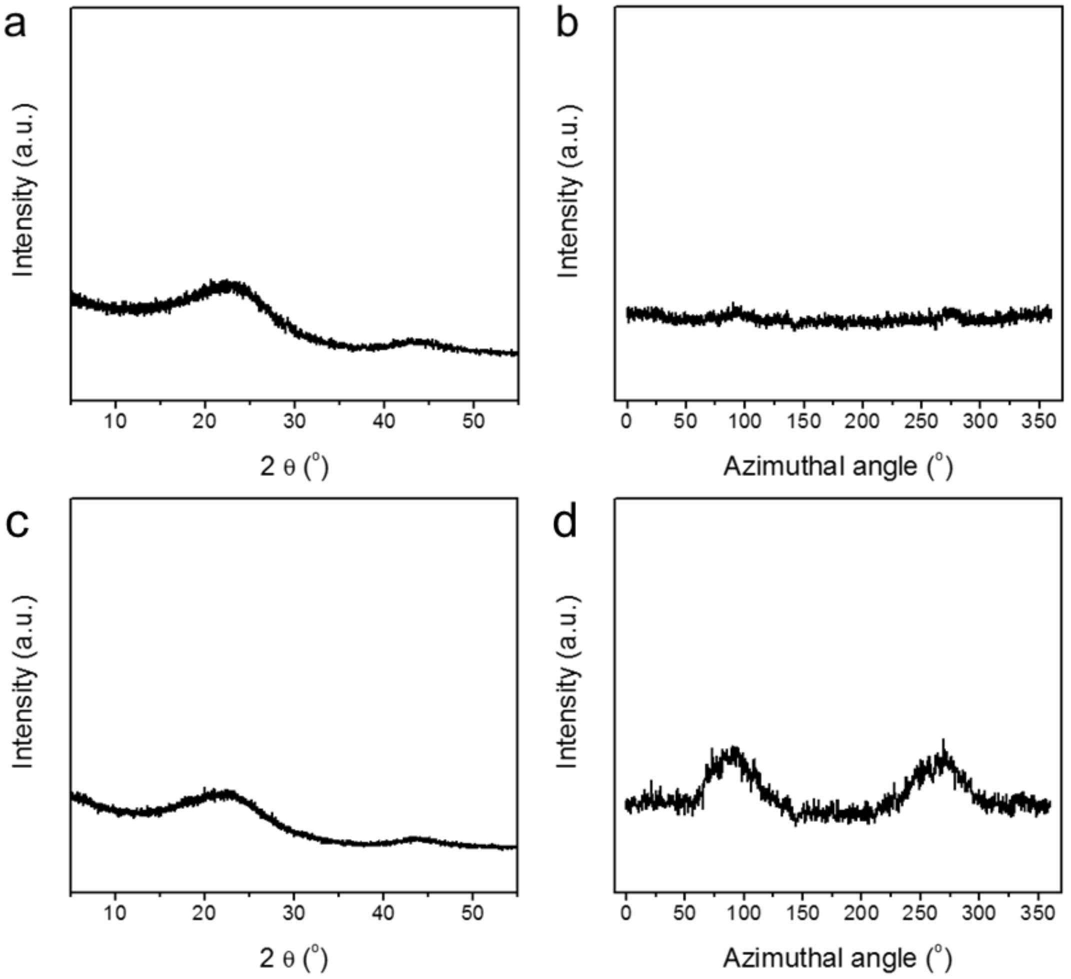

Supplementary Fig. 4. WAXD pattern and 1D azimuthal intensity profile of the radially integrated (002) peak for the silk fibre sample treated without (**a**, **b**) and with (**c**, **d**) applying a loading stress of 7 MPa at 350 °C, in which the β-sheet structure is transformed into an sp^2-hybridised carbon hexagonal structure.

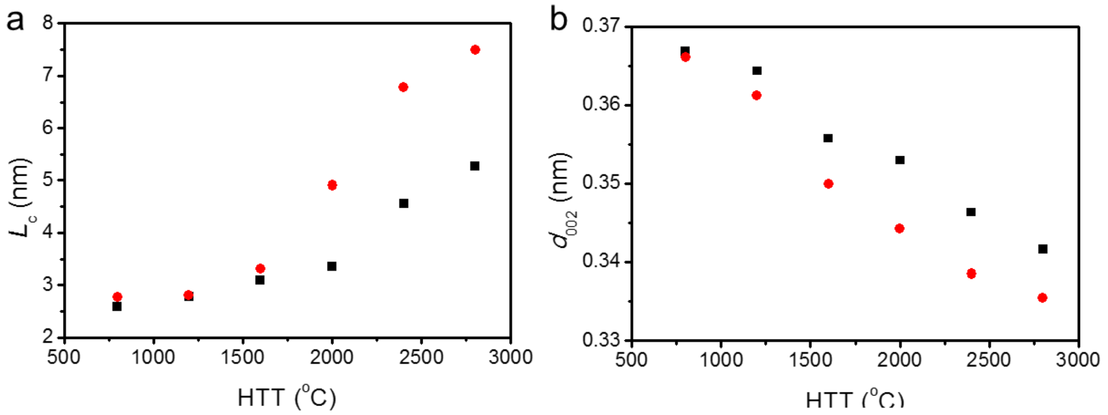

Supplementary Fig. 5. (**a**) Crystallite height (along the c axis, L_c) and (**b**) interlayer spacing (d_{002}) obtained from the XRD (002) peak profiles of silk fibres heated at different temperatures up to 2,800 ºC with (red) and without (black) axial stretching.

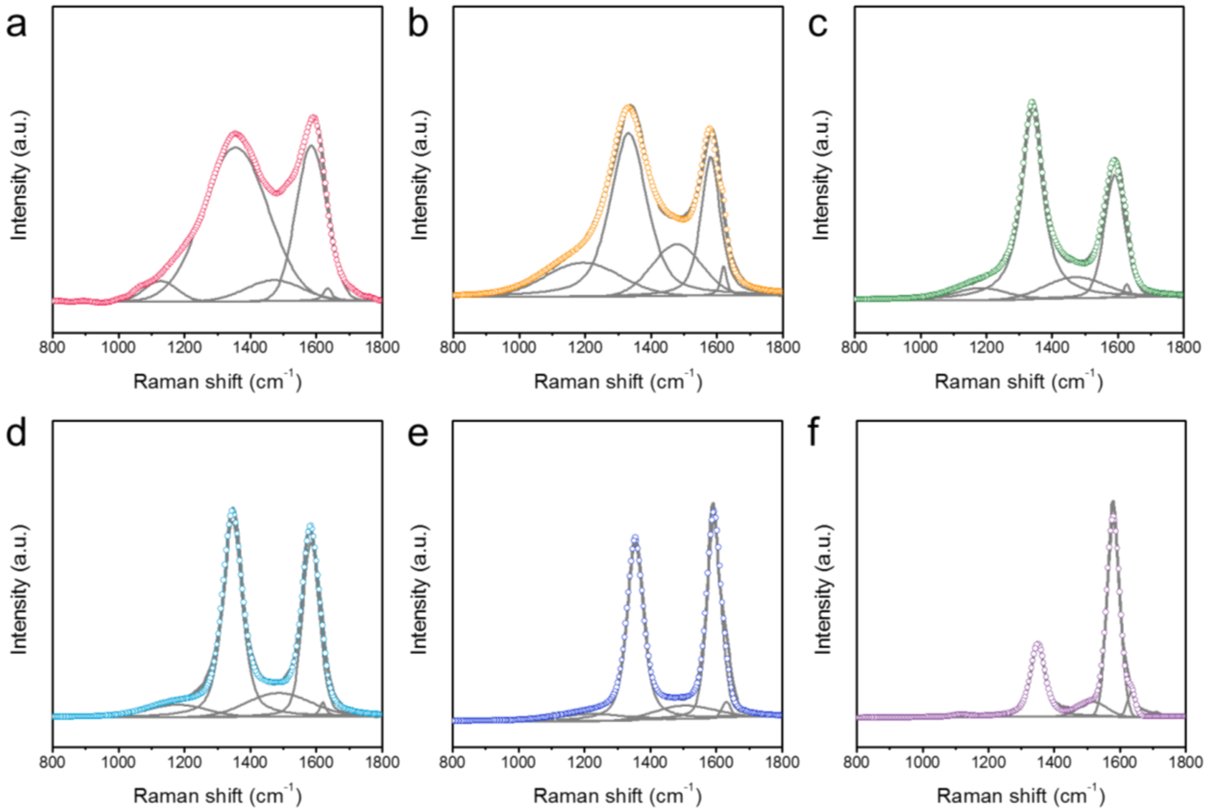

Supplementary Fig. 6. Deconvolved Raman spectra of silk fibres heat-treated at (**a**) 800 ºC, (**b**) 1,200 ºC, (**c**) 1,600 ºC, (**d**) 2,000 ºC, (**e**) 2,400 ºC, and (**f**) 2,800 ºC.

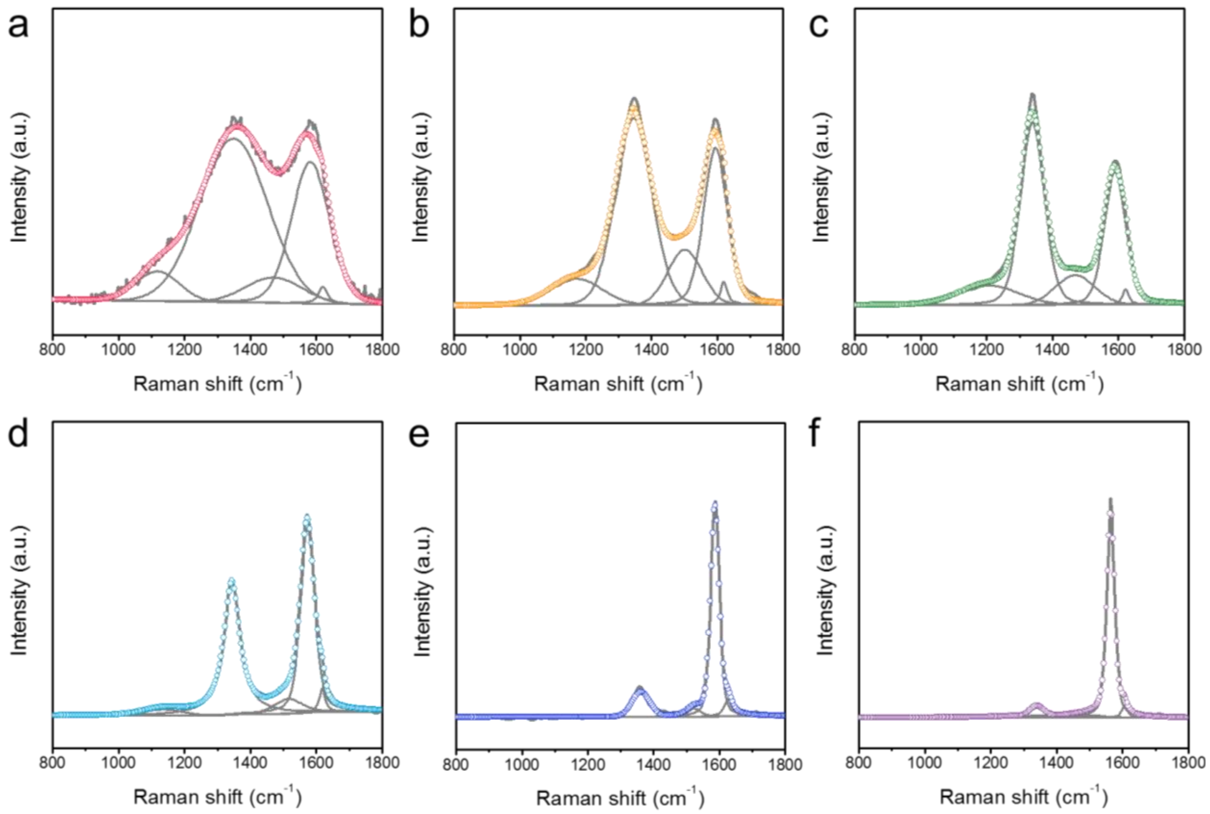

Supplementary Fig. 7. Deconvolved Raman spectra of silk fibres heat-treated at (**a**) 800 ºC, (**b**) 1,200 ºC, (**c**) 1,600 ºC, (**d**) 2,000 ºC, (**e**) 2,400 ºC, and (**f**) 2,800 ºC with axial stretching.

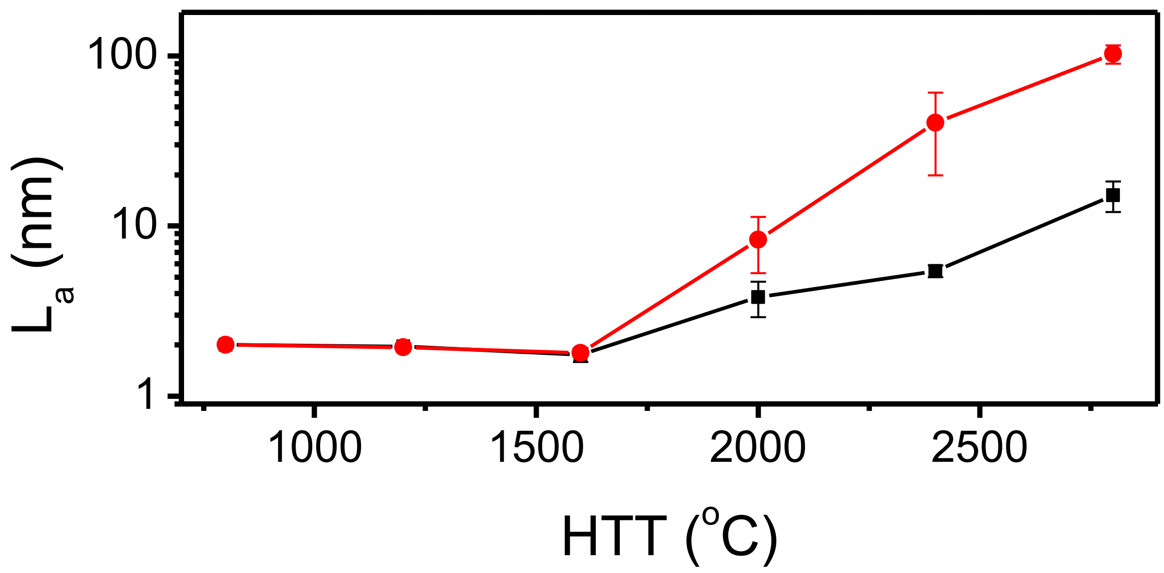

Supplementary Fig. 8. Temperature dependence of the in-plane crystallite sizes, L_a, calculated from Raman spectra of pyroprotein-based fibres heat-treated at different temperatures with (red) and without (black) axial stretching (n=3 for each of the samples). The error bars denote standard deviations of the mean.

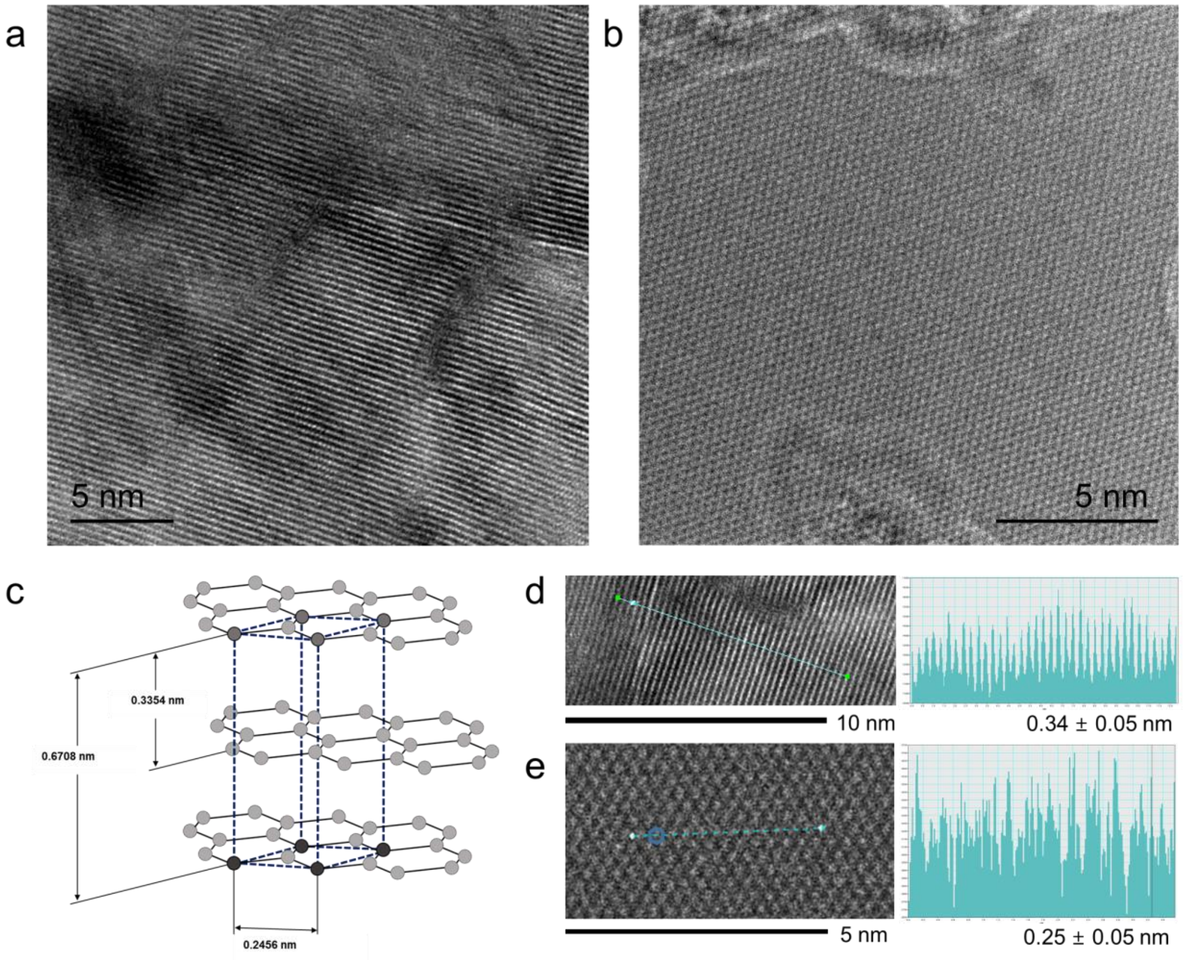

Supplementary Fig. 9. (**a**,**b**) Atomic-scale TEM images of silk fibres heat-treated at 2,800 °C with axial stretching. (**c**) Hexagonal unit cell structure of graphite. Selected images from atomic-scale TEM images (left) and the contrast profiles along the arrows (right) representing (**d**) the interlayer space and (**e**) hexagonal graphitic structure, respectively.

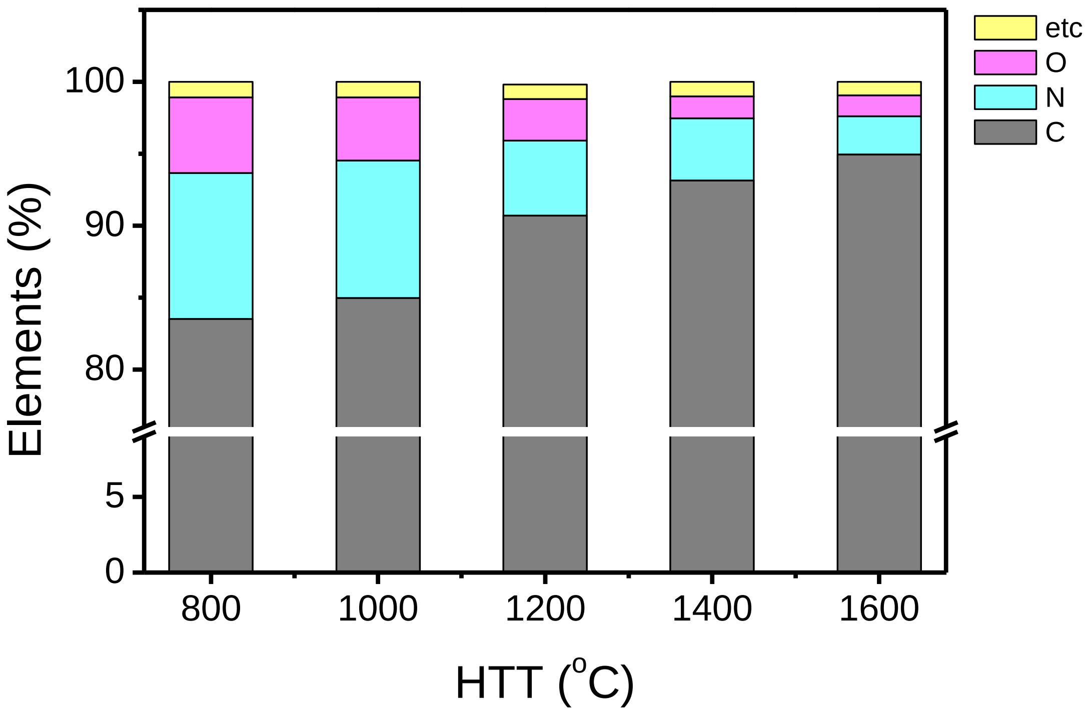

Supplementary Fig. 10. Elemental analysis of the pyroprotein-based fibres with tension as a function of the heat-treatment temperature.

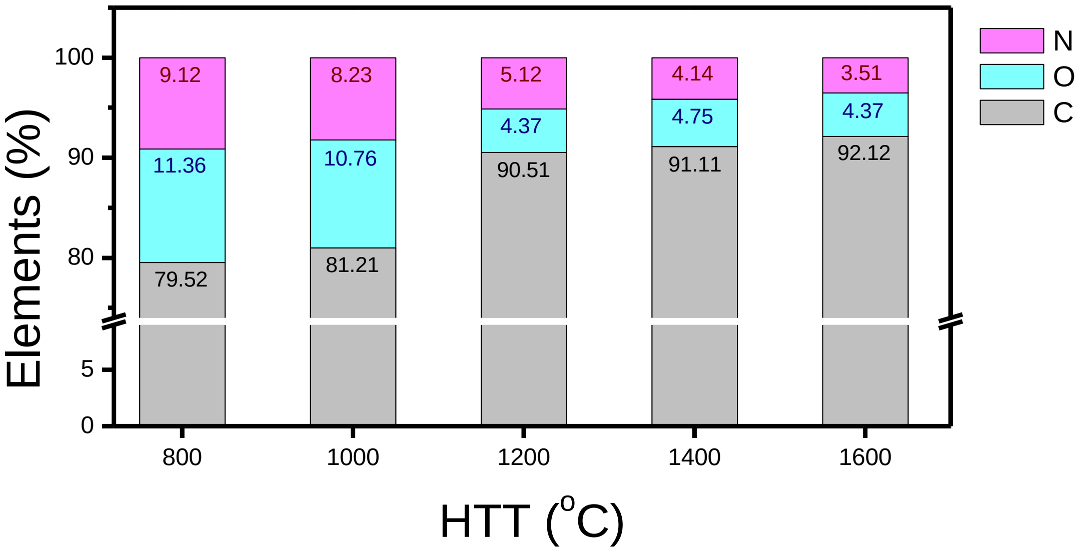

Supplementary Fig. 11. Quantitative X-ray photoelectron spectroscopy results of the pyroprotein-based fibres with tension as a function of the heat-treatment temperature.

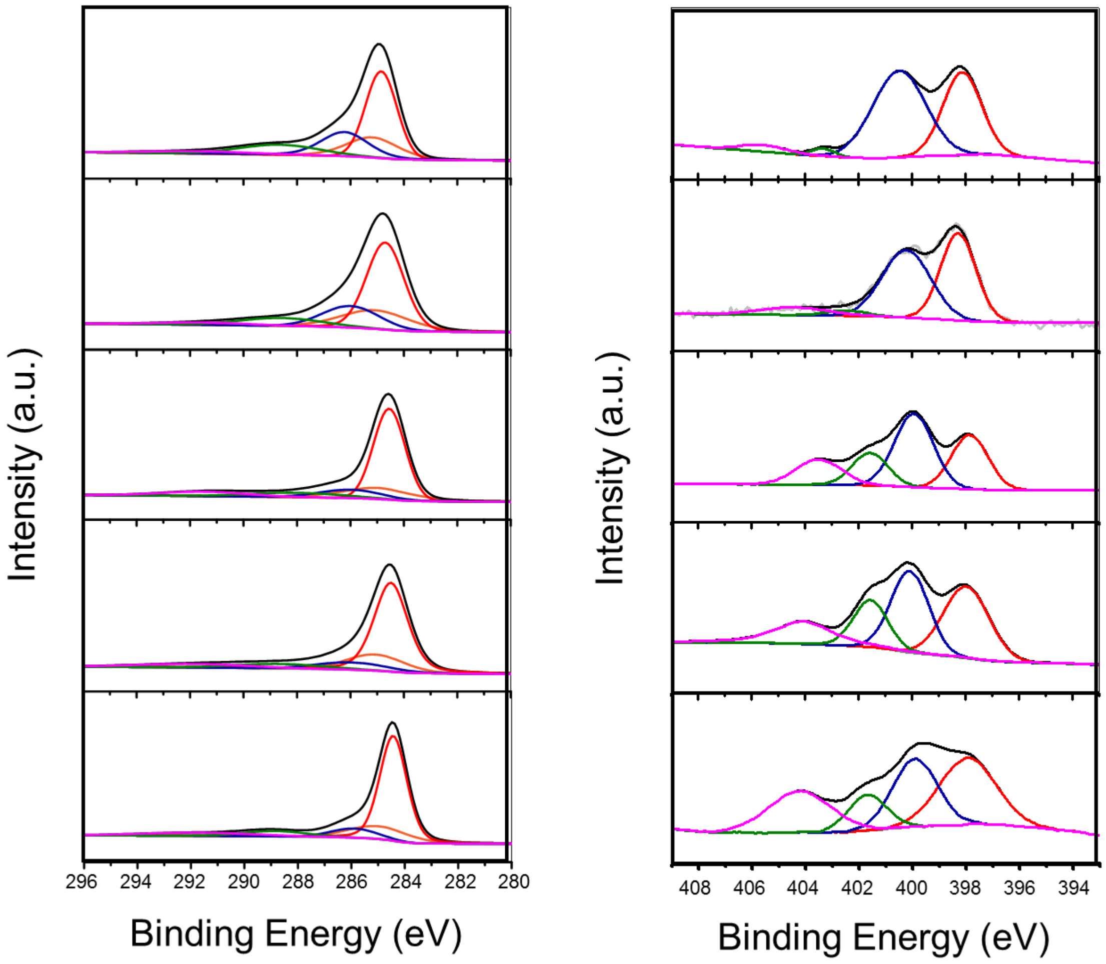

Supplementary Fig. 12. C1s and N1s deconvolution spectra of the pyroprotein-based fibres with tension as a function of the heat-treatment temperature.

Supplementary Table 1

XRD and Raman Parameters.

HTT (℃)	SF			SSF		
	d_{002} (nm)	L_c (nm)	L_a (nm)	d_{002} (nm)	L_c (nm)	L_a (nm)
800	0.3719	0.98	2.01	0.3662	1.01	1.99
1,200	0.3592	1.01	1.96	0.3603	1.12	1.93
1,600	0.3549	1.11	1.75	0.3537	1.16	1.79
2,000	0.3529	1.87	3.81	0.3446	3.32	8.31
2,400	0.3492	2.68	5.43	0.3421	8.69	40.39
2,800	0.3446	4.98	15.14	0.3397	18.98	102.67

Supplementary Table 2

Mechanical and Electrical Properties of the Raw Silk Fibres and Pyroprotein based Fibres.

sample	elongation (%)	tensile strength (GPa)	tensile modulus (GPa)	Density (g/cm^3)	Electrical conductivity (S/cm)
raw silk	15.71±2.46	0.61±0.06	16.22±1.59	1.35	-
SSF800	0.69±0.23	0.79±0.15	77.08±13.55	1.6	-
SSF1000	1.01±0.13	1.49±0.14	129.89±7.89	1.65	-
SSF1200	1.08±0.16	2.48±0.37	244.41±12.27	1.69	8.83×10^2
SSF1400	1.09±0.18	2.57±0.27	248.53±30.75	1.71	9.78×10^2
SSF1600	0.91±0.11	2.35±0.17	255.12±16.25	1.72	1.27×10^3
SSF2000	0.66±0.04	1.94±0.12	310.41±10.51	1.8	1.86×10^3
SSF2400	0.55±0.05	1.89±0.19	377.45±13.46	1.88	2.98×10^3
SSF2800	0.48±0.05	2.02±0.16	472.22±28.05	1.96	4.37×10^3

Supplementary Table 3

N1s deconvolution results

HTT (˚C)	Composition (% of total nitrogen)			
	Pyridinic	Pyridonic/pyrroloc	Quaternary	N-oxide
800	43.45	46.16	2.97	7.42
1000	41.76	42.37	5.05	10.82
1200	27.92	39.83	16.19	16.04
1400	32.19	34.17	14.63	17.01
1600	34.84	30.84	12.76	22.16

Supplementary Table 4

C1s deconvolution results

HTT (˚C)	Composition (% of total carbon)				
	Carbon sp^2	Carbon sp^3	Epoxy/hydroxyl groups(C-O)/C-N	Carbonyl group (C=O)	π–π*
800	47.07	18.63	19.39	10.96	3.95
1000	50.01	18.04	18.15	10.67	3.13
1200	55.84	18.17	10.17	8.23	7.59
1400	59.95	16.45	9.23	7.28	7.09
1600	62.18	15.37	8.46	6.75	7.24

Supplementary Note 1. Native *B. mori* silkworm cocoon fibres (see Supplementary Fig. 1a) are almost pure protein composed of two core microfilaments termed silk fibroin, a glue-like protein (sericin) which holds the silk fibroin together, and some natural impurities[1]. Micro-fibrous proteins with an average diameter of around 10 μm, silk fibroin can be extracted by removing the sericin and impurities through boiling in an alkaline solution (see Supplementary Fig. 1b).

Silk fibroin is composed of three distinct proteins; light chain, heavy chain, and P25 which are present in a 6:6:1 ratio. Although, there are no repetitive sequences in the light chain and P25 encoded as 220 amino acids (Mw ~25 kDa) and 244 amino acids (Mw ~26 kDa), respectively[1-4]. The heavy chain is composed of 5362 amino acids (Mw ~390 kDa) mainly glycine, alanine, and serine up to over 80%[5,6]. The high content of three amino acids result in repeated sequences of short peptides in the primary structure, presented into four modular motifs; two main hexa-peptides including GAGAGS, GAGAGA or GAGYGA (G is glycine, A is alanine, S is serine and Y is tyrosine) and tetra-peptides forming the end of sub-domains such as GAGS or GAAS. Inherently, the protein chains linked by amide bonding are flexible to form diverse molecular conformations. However, numerous supramolecular interactions (such as hydrogen bonding, π-interactions between aromatic groups and van der Waals forces) between the polypeptide chains result in the local conformation (secondary structure) such as α-helices, β-sheets and turns. The highly conserved primary sequence in silk fibroin determines the β-sheet dominant secondary structure. The GX repeats of the amino acid sequence generate hydrogen bonds with each other, and develop to two dimensional chain folding, anti-parallel β-sheet conformation[7]. In addition, amino acids with small side chains such as glycine and alanine, allow inter-sheet stacking into three dimensional nano-crystals by van der Waals forces. A typical β-sheet

crystallite in silk fibroin is known as rectangular with the coordinate system defined with x-axis along the amino acid side chains, y-axis in the direction of the hydrogen bonds, and z-axis along the peptide bonds with lattice constants of a = 0.938 nm, b = 0.949 nm, and c = 0.698 nm (see Supplementary Fig. 1c)[8]. The amorphous regions in silk fibroin composed of non-repeated and hydrophilic amino acids are capable of hydrogen bonding with each other or free water molecules, which enhances the flexibility of silk fibroin by acting as a plasticizer[9]. Thus, the microstructure of native silk fibroin fibers refers to a block copolymer structure of stiff antiparallel β-sheet blocks dispersed in soft amorphous segments and empty space.

In silkworm glands, it is known that the silk fibroins are stored as mesophase intermediates, liquid crystalline. During the spinning process, the silk fibroin molecules are under mechanical forces such as shearing forces from friction caused at the duct wall, extensional forces due to the drawing of the material, and elongational flow, which leads to the formation of crystallite secondary structure and the highly orientated crystallites along the fibre axis (see Supplementary Fig. 1d)[10].

Supplementary Note 2. The change in the diameter and morphology of SSF at various HTTs were investigated using FE-SEM images (Supplementary Figs. 2a-i and Supplementary Fig. 3) and plotted in Supplementary Fig. 4j. After removing the sericins *via* a degumming process, the silk fibroin fibres exhibited an average diameter of 10.4 ± 0.7 μm. After slight shrinkage with a reduction of approximately 2 % before 250 ˚C due to thermal stress, major transverse shrinkage of the SSF samples occurred between 250 and 350 ˚C up to approximately 26 % reduction, attributed to chain scission and the emission of gases resulting from the thermal degradation of the silk protein components. At temperatures in excess of 350°C, the average diameter of the SSF samples gradually decreased as a function of the temperature and the

overall shrinkage is around 43 % for SSF2800. The shrinkage in the longitudinal direction exhibits a similar pattern of transversal shrinkage. After major shrinkage between 250 and 350 ˚C (approximately 17% reduction), the length of the SSF samples gradually decreased as a function of the HTT while retaining their fibrous morphologies.

Supplementary Note 3. The XRD profiles for pyroprotein-based fibres derived from silk are summarized in Supplementary Figs. 5 and Supplementary Table 1 as a function of the HTT. The XRD patterns for all SF and SSF samples have the (002) peak, representing the stacking of carbon layers, which become narrower and shift toward larger angles with increasing HTT, indicating an increase in the number of stacking layers and a decrease in the average interlayer distance[11]. From the XRD results, the carbon crystalline parameter values, the thickness of the crystallites (along the c axis, L_c) and the interlayer distance (d-spacing, d_{002}) are calculated using the Scherrer formula and Bragg's law, respectively, as[12]

$$L_c = K\lambda / \beta\cos\theta \quad (1)$$

$$d_{002} = \lambda / 2sin\theta \quad (2)$$

where K, λ, β, and θ, are the shape factor (normally 0.9), the wavelength of the X-ray source from Cu (1.54 Å), the full width at half maximum in radians, and the diffracted angle, respectively.

Although slightly low values are obtained for the SSF samples, the interlayer spacing of both the SF and SSF ssamples decreases consistently according to the increase in the HTT and gradually approach the value of 0.3354 nm, corresponding to that of ideal graphite[13]. The crystallite thickness, L_c, displays two step growth as a function of the HTT: moderate up to 1,600 °C and fast for higher temperatures especially for the SSF samples (see Supplementary

Fig. 6a and Supplementary Table 1). In general, with increasing HTT, a decrease in the layer distortion is achieved by the elimination of heteroatoms and the thermo-restoration of sp^2 hexagonal carbon layers which allows the distance between the planes to decrease and yields better stacking[14-15].

Supplementary Note 4. Typical Raman spectra of the carbon materials can be classified into first- and second-ordered regions. In the first-ordered region (1,100-1,800 cm^{-1}), there are two representative carbon bands, the E_{2g} vibration mode of graphite layers with sp^2 carbon at a frequency of ～1,580 cm^{-1} (termed the G band) and A_{1g} breathing mode of the sp^2 bonded carbon near the basal edge corresponding to structural defects at a frequency of ～1,350 cm^{-1} (known as the D band)[16,17]. Besides these two major bands, additional smaller bands associated to disordered carbon structures appear at a frequency of around 1,100, 1,500, and 1,620 cm^{-1}. The peaks centered at ~1,100 and ~1,500 cm^{-1} are related with amorphous sp^2 carbons and residual sp^3 carbons, respectively. And the D′ peak centered at ~1,620 cm^{-1} is appeared whenever D band is observed[18]. In the second-ordered region (2,200-3,400 cm^{-1}), the spectra of carbonaceous materials are different for their microstructure. Whereas the disordered carbon materials reveal poorly defined and weak bands at around 2,700 and 2,900 cm^{-1}, the ordered structural carbon materials exhibit well defined band at 2,690 cm^{-1}, named 2D band indicating 3D ordered carbon structure[19], and additional smaller bands centered at 2,450 and 3,200 cm^{-1}.

For the SF800, SF1200, and SF1600 (See Fig. 2e), although the frequencies of D and G bands are relatively high, other disordered carbon characteristic peaks (at around 1,100, 1,500, and 1,620 cm^{-1}) are strongly overlapped in the first-ordered spectrum with no identifiable peaks in the second-ordered region. As the HTT increases to 2,000 ˚C, both D and G bands become sharper and 2D peak centered at 2,690 cm^{-1} is appeared, which corresponds to the development

of an sp^2-hybridized carbon structure and the formation of three-dimensional-ordered stacked graphitic layers, respectively. At higher HTTs, D and G bands further narrow. The G band increases in intensity with increasing temperature, whereas for the D band, the intensity decrease. For the SSF samples, similar Raman spectra were observed for the low HTT cases including SSF800 and SSF1200. However, from 1,600 ˚C, slightly different Raman spectrum are revealed compared to those of SFs. For SSF1600, D and G bands are much sharper and narrower than those of SF1600 and 2D band is shown even somewhat broad, which indicates the development of more ordered structure at relatively low temperature. As the HTT increases to higher temperature, the tendency is more pronounced. Much smaller D band and sharper G band with increased intensity are shown for SSF2000, SSF2400, and SSF2800 compared to those of SF2000, SF2400, and SF2800, respectively. Particularly, for SSF2400 and SSF2800, strong decrease in intensity of D band and weakening of the bands at ~2,900 and ~3,200 cm^{-1}, indicating the development of triperiodic graphite structure.

Crystalline sp^2 carbon clusters are often defined in terms of their parallel average size, L_a, which can be calculated from the ratio of Raman intensities of D and G bands, I_D/I_G. When $L_a > 2$ nm, Tuinstra & Koenig (TK) equation was used as shown by[20]:

$$I_D/I_G = C(\lambda)/L_a \qquad (3)$$

where C(λ) is a constant dependent on the laser wavelength (4.4 for a 514 nm laser here). In case of $L_a < 2$ nm, Ferrari & Robertson (FR) relationship was utilized following as[16]

$$I_D/I_G = C'(\lambda)/L_a^2 \qquad (4)$$

where C'(λ) is represents a wavelength-dependent prefactor calculated by the following relation:

$$C'(\lambda) = C_0 + \lambda C_1 \qquad (5)$$

Where, C_0 = -12.6 nm and C_1 = 0.033[21,22].

In order to estimate the ratio of I_D/I_G of SFs and SSFs, First ordered regions of Raman spectra were deconvolved using five peaks at 1100, 1350, 1500, 1580 and 1620 cm^{-1} (See Supplementary Figs 6 and 7). Both SFs and SSFs show almost constant I_D/I_G ratio up to 1,600 ˚C with a value of ~ 2, corresponding to ~2 nm of L_a. At higher temperature above 2,000 ˚C, I_D/I_G ratio of both SF and SSF samples become lower (indicating the expansion of carbon clusters) at different rates. For SF2000, SF2400, and SF2800, 0.94, 0.81, and 0.31 of I_D/I_G ratios were estimated, corresponding to 3.81, 5.43, and 15.14 nm of L_a, respectively. The evolution of carbon layer according to HTTs are similar to the typical non-graphitizable carbon materials reported elsewhere[23]. However, in case of SSFs, substantially low I_D/I_G ratios were observed for SSF2000, SSF2400, and SSF2800 with 0.61 0.11, and 0.04 corresponding to 8.31, 40.39, and 102.67 nm of L_a, respectively (See Supplementary Fig. 8), almost similar to graphitizable carbon materials[13].

Supplementary Note 5. FE-TEM micrographs and selected area diffraction patterns (SAED) were obtained from the thinnest fragments from the finely ground SF and SSF samples heat treated to various temperatures between 800 and 2,800 ˚C as shown in Figs. 3c,d). FE-TEM images of SF samples resembles a typical amorphous carbon phase, observed for organic materials. By 800 ˚C, SF reveal no distinctive structure with a lot of disordered voids. With increasing HTTs up to 1,600 ˚C, even the nano-metric carbon crystallites are observed, they are disordered and only localized, corresponding to the SAED patterns showing faint and broad 002 rings. By further heating up to 2,800 ˚C, the carbon cluster expanded into the long-range carbon layers with curvature, resulting few-layer-stacking as shown in the XRD and Raman data. However, the structural evolution of SSFs is not in agreement with typical organic

materials. For SSF800, there is no long-range order, and some regions consist of aligned carbon layers corresponding to the 002 arcs and faint 10 and 11 lines within the diffraction pattern. With as the HTT increase to 1,600 ˚C, the parallel orientation of the carbon layers (and of the pore walls) along the fibre axis improves and the interlayer spacing becomes smaller and more constant. For SSF2000, the layers become more planar and the length of the carbon layers starts to increase. In the corresponding SAED pattern, 004 orders appear, which indicates that the carbon layers become less distorted. SSF2400 shows a dramatic structural change with the long-range ordered structure with some residual misoriented stacks of layers. At the highest temperature, 2800 C, the FE-TEM image shows well-defined layers of long-range order stacked parallel to each other, corresponding to the SAED pattern with weak 10 and 11 bandmodulations indicating the transition from a two-dimensional turbostratic structure to a three-dimensional crystalline structure.

Supplementary Note 6. Elemental analysis was performed to estimate the chemical evolution of the SSF samples as a function of HTT (Supplementary Fig. 10). According to the increase of HTT, the chemical compositions clearly indicate an enrichment of the carbon fraction with the decrease of the nitrogen and oxygen content. This trend was also observed by XPS measurements, even the atomic percentages between both techniques slightly different because of the technical differences (Supplementary Fig. 11). Four peaks were proposed from the deconvolution of the N 1s peak (Supplementary Fig. 12 and Supplementary Table 3): pyridinic, pyridonic/pyrrolic, quaternary and oxidized nitrogens, with binding energies at ~398.5, ~401.0, ~401.5, and ~403.5 eV, respectively. The amount of pyridinic N and pyridonic/pyrrolic N decreased, bur the amount of oxidized N increased with an increase of HTT. In case of the quaternary nitrogen, the fraction becomes a maximum at the carbonization temperature of

1,200 °C, and starts to decrease by further heating. Furthermore, from the C 1 s deconvolution, we observed that the amount of sp^3 also starts to decrease from 1,200 °C (Supplementary Fig. 12 and Supplementary Table 4). Consequently, the carbonization temperature of 1,200 °C results in maximum fractions of both sp^3 and quaternary nitrogen content, leading to higher tensile strength even though the carbonization temperature is different.

Supplementary References

1. Rockwood, D. N. *et al*. Materials fabrication from Bombyx mori silk fibroin." *Nature protocols* **6**, 1612-1631 (2011).
2. Asakura, T. *et al*. The role of irregular unit, GAAS, on the secondary structure of Bombyx mori silk fibroin studied with 13C CP/MAS NMR and wide-angle X-ray scattering. *Protein Sci.* **11**, 1873-1877 (2002).
3. Vepari, C. & Kaplan, D. L. Silk as a biomaterial. *Prog. Polym. Sci* **32**, 991-1007 (2007).
4. Inoue, S. *et al*. Silk fibroin of Bombyx mori is secreted, assembling a high molecular mass elementary unit consisting of H-chain, L-chain, and P25, with a 6: 6: 1 molar ratio. *J. Biol. Chem.* **275**, 40517-40528 (2000).
5. Marsh, R. E., Corey, R. B., & Pauling, L. The structure of tussah silk fibroin (with a note on the structure of β-poly-l-alanine)." *Acta Crystallogr.* **8**, 710-715 (1955).
6. Tristram, G. R., & Smith, R. H. The amino acid composition of some purified proteins. *Adv. Protein Chem.* **18**, 227-318 (1964).
7. Hardy, J. G., Römer, L. M., & Scheibel, T. R. Polymeric materials based on silk proteins. *Polymer* **49**, 4309-4327 (2008).
8. Drummy, L. F., Farmer, B. L., & Naik, R. R. Correlation of the β-sheet crystal size in silk fibers with the protein amino acid sequence. *Soft Matter* **3**, 877-882 (2007).
9. Altman, G. H. *et al*. Silk-based biomaterials. *Biomaterials* **24**, 401-416 (2003).
10. Walker, A. A., Holland, C., & Sutherland, T. D. More than one way to spin a crystallite: multiple trajectories through liquid crystallinity to solid silk. *Proc. R. Soc. B* **282**, 20150259 (2015)

11. Li, Z. Q. *et al*. X-ray diffraction patterns of graphite and turbostratic carbon. *Carbon* **45**, 1686-1695 (2007).

12. Short, M. A., & Walker, P. L. Measurement of interlayer spacings and crystal sizes in turbostratic carbons. *Carbon* **1**, 3-9 (1963).

13. Vázquez-Santos, M. B. *et al*. Comparative XRD, Raman, and TEM study on graphitization of PBO-derived carbon fibers. *J. Phys. Chem. C* **116**, 257-268 (2011).

14. Krause, S. J. et al. Morphology and properties of rigid-rod poly (p-phenylene benzobisoxazole)(PBO) and stiff-chain poly (2, 5 (6)-benzoxazole)(ABPBO) fibres. *Polymer* **29**, 1354-1364 (1988).

15. Edstrom, T., & Lewis, I. C. Chemical structure and graphitization: X-ray diffraction studies of graphites derived from polynuclear aromatics. *Carbon* **7**, 85-91 (1969).

16. Ferrari, A. C., & Robertson, J. Interpretation of Raman spectra of disordered and amorphous carbon. *Phys. Rev. B* **61**, 14095 (2000).

17. Ferrari, A. C., & Robertson, J. Resonant Raman spectroscopy of disordered, amorphous, and diamondlike carbon. *Phys. Rev. B* **64**, 075414 (2001).

18. Jawhari, T., Roid, A., & Casado, J. Raman spectroscopic characterization of some commercially available carbon black materials. *Carbon* **33**, 1561-1565 (1995).

19. Dresselhaus, M. S. *et al*. Perspectives on carbon nanotubes and graphene Raman spectroscopy. *Nano lett.* **10**, 751-758 (2010).

20. Tuinstra, F., & Koenig, J. L. Raman spectrum of graphite. *The Journal of Chemical Physics* **53**, 1126-1130 (1970).

21. Zickler, G. A., *et al*. A reconsideration of the relationship between the crystallite size L

a of carbons determined by X-ray diffraction and Raman spectroscopy. *Carbon* **44**, 3239-3246 (2006)

22. Matthews, M. J. *et al*. Origin of dispersive effects of the Raman D band in carbon materials. *Phys. Rev. B* **59**, R6585 (1999).

23. Ōtani, S. On the carbon fiber from the molten pyrolysis products. *Carbon* **3**, 31IN335-3438 (1965).
